# The effects of an 8-week functional training program on functional movement and physical fitness in male university students: a randomized controlled trial

**DOI:** 10.3389/fpubh.2025.1641590

**Published:** 2025-08-29

**Authors:** Lingwei Li, Fanyi Meng, Siqi Yao, Qingfu Shi, Chao Luo

**Affiliations:** ^1^College of Physical Education, Zhengzhou University of Light Industry, Zhengzhou, China; ^2^School of Education, Yangtze University, Jingzhou, China; ^3^School of Physical Education, Zhengzhou University, Zhengzhou, China; ^4^College of Sports Science, Jishou University, Xiangxi, China; ^5^Changsha Commerce & Tourism College, Changsha, China

**Keywords:** functional movement screen (FMS), core stability, movement patterns, physical education, injury prevention

## Abstract

**Objective:**

The physical fitness and functional movement capabilities of university students are fundamental to their long-term health, yet traditional physical education models may have limitations in concurrently enhancing both aspects. This study aimed to compare the effects of an 8-week functional training (FT) program versus a traditional training (TT) program on functional movement quality and comprehensive physical fitness in non-athlete male university students.

**Methods:**

This study was a randomized controlled trial. Thirty-five healthy male university students (mean age 18.8 ± 0.9 years) were randomly assigned to either a functional training group (FT group; *n* = 20) or a traditional training group (TT group; *n* = 20). During the 8-week intervention, the FT group performed exercises centered on integrated, multi-joint, multi-planar movements. The TT group engaged in a traditional physical education program of matched duration and frequency, which focused on isolated fitness exercises and basic sports skills. Assessments were conducted at baseline (week 0), mid-intervention (week 4), and post-intervention (week 8). Main outcome measures included scores for the seven components of the Functional Movement Screen (FMS) and five physical fitness indicators (50-m sprint, 1,000-m run, sit-and-reach, standing long jump, and vital capacity).

**Results:**

Significant time × group interaction effects were observed for most outcome measures. Compared to the TT group, the FT group demonstrated significantly greater improvements in the total FMS score and several components, particularly those assessing dynamic stability and core control (e.g., Active Straight-Leg Raise, Rotary Stability; *p* < 0.01). Furthermore, the FT group showed superior gains across all five physical fitness indicators, including speed, endurance, flexibility, power, and vital capacity (all interaction effects, *p* < 0.05).

**Conclusion:**

For male university students, an 8-week functional training program is more effective than a traditional training regimen for concurrently improving functional movement patterns and comprehensive physical fitness. These findings support the integration of functional training principles and methods into university physical education curricula to more effectively enhance students’ overall physical preparedness and movement quality.

## 1 Introduction

The university period represents a critical juncture in an individual’s life, significantly influencing the establishment of long-term health behaviors and overall well-being (1). Adequate physical fitness and proficient functional movement capabilities are paramount for university students, not only contributing to enhanced academic performance and mental health (2) but also laying the foundation for a reduced risk of chronic diseases in later life (3). However, contemporary university populations often exhibit concerning trends toward sedentary lifestyles and a decline in overall physical conditioning (4), highlighting an urgent need for effective physical education and training interventions. Traditional physical training programs within university settings have often emphasized sport-specific skills or isolated fitness components, such as cardiovascular endurance or muscular strength in specific muscle groups (5). While beneficial to a certain extent, these conventional approaches may not adequately address the holistic development of functional movement patterns, core stability, and neuromuscular coordination, which are essential for optimal physical function and resilience (6). In response to these limitations, Functional Training (FT) has emerged as a comprehensive training paradigm. Functional Training is defined as a system of training that prepares the body for the demands of real-life activities and sports, focusing on multi-joint, multi-planar, integrated movement patterns rather than isolated muscle actions (7). It is crucial, however, to distinguish FT from other dynamic training modalities to clarify its unique contribution. For instance, while plyometric training specifically targets the enhancement of explosive power via the stretch-shortening cycle, FT adopts a broader scope by integrating power development with stability, balance, and multi-planar control, focusing on the proficient execution of a complete movement pattern rather than isolated power output (8). Similarly, whereas circuit training defines a structural workout format—moving between exercises with minimal rest primarily for metabolic conditioning—FT represents a training philosophy that dictates the *selection* of exercises (9). An FT session may be structured as a circuit, but its defining characteristic is the prioritization of integrated movements that improve neuromuscular coordination and real-world function. The unique contribution of FT, therefore, lies in its primary focus on improving the *quality and efficiency* of foundational movement patterns, making it a distinct approach to physical conditioning.

Despite the growing popularity and distinct philosophy of FT, there remains a need for robust empirical evidence comparing its efficacy against traditional training methods in improving both functional movement competency and comprehensive physical fitness among university students. While some studies have explored FT in athletic or clinical populations, its specific impact on this broader demographic requires further elucidation, particularly within the context of modern screening and prevention paradigms. In recent years, composite indices and test batteries have been developed to comprehensively evaluate physical fitness and identify health risks in youth and young adults. For instance, the ASSO-Fitness Test Battery, part of the Adolescents and Surveillance System for the Obesity prevention (ASSO) project, provides a validated Fitness Index model based on multiple components, including the standing broad jump (10). Such models underscore the importance of multi-dimensional assessments for establishing performance benchmarks and identifying at-risk populations.

Aligning with this comprehensive assessment philosophy, the present study employs a dual-pronged evaluation strategy. We utilize the FMS, a widely used tool to assess the quality of fundamental movement patterns, identify limitations, and screen for potential injury risk (11). Simultaneously, we employ a battery of health-related fitness tests (50-m sprint, 1,000-m run, etc.) to quantify the capacity of different physiological systems. This combined approach provides a more holistic evaluation than either assessment would in isolation, allowing for an understanding of not just how much an individual can do (i.e., their physical capacity), but also how well they move (i.e., their movement quality). Therefore, a key contribution of this research is to investigate how a targeted intervention like FT affects both of these critical dimensions of physical preparedness concurrently.

## 2 Subjects and methods

### 2.1 Experimental subjects

To guarantee adequate statistical power for testing the core hypothesis, we did *a priori* power analysis with GPower (Version 3.1) before the experiment. The analysis focused on the time-group interaction effect in Repeated Measures ANOVA to determine the required sample size. We drew on the conventions in related fields for the parameters. They included a medium effect size (*f* = 0.25), *α* = 0.05, and power (1 − β) = 0.80. Other parameters were two groups, three repeated measures, and an estimated correlation among repeated measures of 0.5. Given these, GPower indicated a minimum of 34 participants. Our study recruited 40 valid participants, satisfying and slightly exceeding the calculated requirement. This shows the study has sufficient statistical power to test the main hypothesis.

This 8-week parallel – group, randomized controlled trial compared the effects of functional training and traditional training on the physical fitness of male college students. The study was approved by the university’s ethics committee, and all participants gave written informed consent after understanding the study details. Fourty healthy male students (mean age 18.8 ± 0.9 years) were recruited. Inclusion criteria: non-sports majors with no systematic sports training in the past 6 months. Exclusion criteria: musculoskeletal injuries, cardiopulmonary diseases, or chronic conditions affecting training. The basic information is shown in Table 1.

**Table 1:** Demographic characteristics of participants.

Parameter	Control group	Experimental group	***p***-value
Age (y)	19.00 ± 0.926	18.60 ± 0.986	0.724
Height (cm)	177.67 ± 4.546	176.33 ± 6.040	0.714
Body mass (kg)	69.00 ± 4.598	65.00 ± 8.534	0.359

Statistical analysis revealed no significant intergroup differences in age, height, or body mass (*p* > 0.05), confirming cohort homogeneity for experimental requirements.

Participants were asked to maintain their usual lifestyle and diet and avoid extra structured sports training during the study. After baseline tests, a research assistant not involved in the study used a computer-generated random sequence to allocate 40 participants into the functional training group (FT, *n* = 20) and traditional training group (TT, *n* = 20). Allocation results were in opaque sealed envelopes to prevent predetermination or influence. Blinding participants and trainers wasn’t feasible due to the intervention nature, but participants were only told that two training methods would be compared. Assessors were blinded to group allocation and did not participate in training sessions to prevent measurement bias. This study confirms that all studies were conducted in accordance with relevant guidelines/regulations, and that informed consent forms were obtained from all participants and/or their legal guardians. This study was conducted strictly in accordance with the Helsinki Declaration. This study obtained ethical approval from Zhengzhou University of Light Industry Ethics Committee (Protocol No: 2024-FT-018).

### 2.2 Experimental scheme and implementation steps

1.  Experimental time and cycle: from October 2024 to December 2024 in the stadium of Zhengzhou University of light industry. There were 8 weeks in total. The first week was the preparation for the experiment, the sixth and eleventh weeks were the test, and the experiment lasted for 8 weeks; The training time of the experimental group is every Tuesday morning, and the training time of the control group is every Friday morning. The duration of each training is 90 min.
2.  To ensure the objectivity and consistency of the functional movement screen (FMS) scoring, strict quality control measures were implemented in this study. All assessments were conducted by a single, experienced examiner who was FMS Level 2 Certified.

Prior to the formal commencement of the experiment, a pilot study was conducted to establish rater reliability and minimize potential subjective scoring bias. The specific procedures were as follows: Examiner Training and Standardization: The designated examiner underwent a refresher training course on the standardized FMS testing procedures and scoring criteria to ensure a thorough and consistent understanding of the 0–3 point scale for each movement.

Reliability testing: A sample of 10 volunteers, who were not part of the main study, were recruited and video-recorded while performing the FMS tests. The study’s examiner independently scored these 10 video samples on two separate occasions, with a one-week interval between ratings. This “blinded” re-scoring (i.e., the rater was unaware of the initial scores) was used to calculate the intra-rater reliability using the Intraclass Correlation Coefficient (ICC). Furthermore, the examiner’s initial scores were compared against those of a senior FMS expert (serving as the gold standard) to assess inter-rater reliability.

The results of the pilot study indicated excellent reliability. The examiner’s intra-rater reliability ICC was 0.92, and the inter-rater reliability ICC was 0.89. Both values are well above the threshold for excellent agreement (ICC > 0.80). These findings confirm that the FMS examiner for this study possessed a high degree of stability and reliability in scoring, thereby ensuring the accuracy and credibility of the collected FMS data.

3.  This study was conducted as a randomized controlled trial. Following baseline assessments and obtainment of informed consent from all participants, a rigorous randomization and allocation concealment procedure was implemented. Specifically, a research assistant independent of the study team (i.e., not involved in participant recruitment, training instruction, or outcome assessment) used a computer-generated random number sequence to assign the 40 participants to either the functional training group (FT group; *n* = 20) or the traditional training group (TT group; *n* = 15) at a predefined ratio. The allocation for each participant was placed in a sealed, opaque envelope. The respective training instructors opened the envelopes to reveal the group assignment only immediately before the first training session, ensuring that neither the researchers nor the participants could foresee or influence the group allocation process.

Given the nature of the physical training interventions, blinding of the participants and training instructors was not feasible. However, the following measures were taken to minimize potential bias:1. Participant Information Control: All participants were informed that the study aimed to “compare the effects of two different physical training methods on physical fitness,” but they were kept unaware of the specific research hypotheses (i.e., which method was presumed to be superior) and the theoretical differences between the training protocols.2. Assessor Blinding: To eliminate detection bias, the outcome assessor responsible for conducting all Functional Movement Screen (FMS) and physical fitness tests was blinded to the group assignments of the participants throughout the entire study period (from baseline to final assessment). This assessor was not involved in any training instruction, and all data were coded (e.g., Participant A, B, C) prior to analysis to ensure the objectivity of the outcome evaluation.

4.  To ensure the objectivity and consistency of the functional movement screen (FMS) scoring, strict quality control measures were implemented in this study. All assessments were conducted by a single, experienced examiner who was FMS Level 2 Certified.

Prior to the formal commencement of the experiment, a pilot study was conducted to establish rater reliability and minimize potential subjective scoring bias. The specific procedures were as follows:

Examiner training and standardization: The designated examiner underwent a refresher training course on the standardized FMS testing procedures and scoring criteria to ensure a thorough and consistent understanding of the 0–3 point scale for each movement.

Reliability testing: A sample of 10 volunteers, who were not part of the main study, were recruited and video-recorded while performing the FMS tests. The study’s examiner independently scored these 10 video samples on two separate occasions, with a one-week interval between ratings. This “blinded” re-scoring (i.e., the rater was unaware of the initial scores) was used to calculate the intra-rater reliability using the Intraclass Correlation Coefficient (ICC). Furthermore, the examiner’s initial scores were compared against those of a senior FMS expert (serving as the gold standard) to assess inter-rater reliability.

The results of the pilot study indicated excellent reliability. The examiner’s intra-rater reliability ICC was 0.92, and the inter-rater reliability ICC was 0.89. Both values are well above the threshold for excellent agreement (ICC > 0.80). These findings confirm that the FMS examiner for this study possessed a high degree of stability and reliability in scoring, thereby ensuring the accuracy and credibility of the collected FMS data.

5.  Design of functional training program: the 8-week functional training is designed in stages (please refer to Table 2 for specific details): the first link (warm-up activation), which is activated by dynamic stretching to improve muscle flexibility and nerve control ability; The second link (main body training), carry out targeted main body training, including speed endurance training and functional strength training, in order to enhance students’ physique; The third link (relaxation and stretching), through jogging and systematic static stretching (covering the main muscle groups of chest and shoulder, neck and back, trunk and lower limbs), is to sort out and relax, promote recovery and reduce the risk of sports injury. The whole program focuses on action function and muscle coordination, aiming to comprehensively improve students’ sports performance and health level. The following is the specific content of the training plan. Each functional training plan selects 2–4 actions from the three links of warm-up activation, main body training, relaxation and stretching in the table below to further arrange reasonable classroom exercises.
6.  Implementation steps: the experimental group was trained with functional training methods, and the control group was trained with traditional sports training methods. There is no other sports participation outside the training time, and the training exercise volume, exercise load and exercise intensity are consistent. Before training, the experimental group and the control group were tested for FMS and Physical Fitness, and the pre-test experimental data were obtained. After the experiment of the two training methods, the scores of the two groups were retested, and the test scores were statistically analyzed by SPSS 22.0.
7.  The training session commenced with a 15-min warm-up phase designed to elevate heart rate and muscle temperature. This phase included a 2- to 3-lap jog around a standard 400-m athletics track (approximately 800–1,200 m), followed by basic dynamic stretches and joint mobility exercises such as high knees, butt kicks, arm circles, static lunges, and trunk rotations. The main training phase, lasting 65 min, was composed of three distinct modules. The first module, a 20-min cardiorespiratory endurance session, required participants to complete four sets of 400-m interval runs on the track at an intensity of approximately 70–80% of their maximum effort, with 2 to 3 min of slow walking or complete rest between sets. The second module was a 25-min strength conditioning session focusing on fundamental bodyweight exercises for major muscle groups, which included three sets of push-ups (performed to near-failure), three sets of 20–30 sit-ups, three sets of 15–20 parallel-stance bodyweight squats, and three sets of 45–60 s planks. The final module of the main phase consisted of 20 min of basic ball skills practice, using basketball as an example, which covered stationary dribbling with alternating hands, two-person chest and bounce passes, and set shooting practice near the free-throw line. The final 10 min of the session were dedicated to a cool-down phase, during which participants performed one lap of slow walking followed by systematic static stretching for all major muscle groups, holding each stretch for 20 to 30 s.

**Table 2:** Functional training protocol.

Exercise	Movement standard	Key execution cues	Sets × Reps/Duration
Knee-to-chest walk	Alternating knee elevation with manual assistance	Maintain erect torso, engage core	2 × 20 m
Lunge with rotation	Rotational reach in split stance	Stabilize pelvis, prevent knee valgus	8–10/side × 2
High-knee run	Rapid knee drive to hip height	Upright posture, forefoot strike	30s × 2–3 (20s rest)
Lateral shuffle	Low-center-of-gravity side steps	Toe forward, decelerate controlled	10/steps/side × 2
Plyometric footwork	Rapid treading → abrupt stop	Knee aligned over 2nd toe	10s + 3 stops × 3–4
8-Level plank progression	Full-body alignment maintenance	Prevent lumbar hyperextension/rotation	2–3 (60s rest)
Quadrupedal crawling	Cross-limb contralateral pattern	Slow tempo for core activation	10 steps (fwd + bwd) × 2
Multi-directional sprints	Forward/backward/lateral acceleration	Lower center of gravity	3 × 2 lengths
Med ball slam → Sprint	Overhead slam (4–6 kg) → 15 m sprint	Hip-driven power transfer	8–10 × 3 (45 s rest)
Single-leg RDL	Controlled hip hinge	Neutral spine, slight knee flexion	8–10/side × 3
Bulgarian split squat	Rear-elevated unilateral squat	Heel weight distribution	8–10/side × 3
Box jump	Vertical propulsion → soft landing	Arm swing contribution	6–8 × 3 (60s rest)
Lateral bounding	Single-leg side jumps with pause	Forefoot absorption	6–8/side × 3
Ladder drills	Low-amplitude foot patterns	Horizon-focused gaze	3 × 2 passes
Cat-cow stretch	Segmental spinal articulation	Controlled breathing cycles	8 breaths/phase × 2
Foam rolling	Myofascial release on major muscles	Circumvent bony prominences	30s/muscle group

This control group protocol differed significantly from the functional training group’s program in its core philosophy. Its content was characterized by a modular and isolated approach, wherein fitness components such as cardiorespiratory endurance, strength, and skills were trained separately, whereas the experimental group’s functional training emphasized integrated, multi-joint, multi-planar movement patterns, focusing on movement quality and synergistic body control. In terms of exercise selection, the control group predominantly utilized traditional, linear, single-plane exercises such as sit-ups and conventional squats, while the experimental group incorporated more complex movements involving instability, asymmetry, and rotation, for instance, Bulgarian split squats, lunges with rotation, and medicine ball slams. Furthermore, regarding recovery methods, the control group primarily employed traditional static stretching techniques, while the experimental group integrated more contemporary methods like foam rolling for myofascial release to promote deeper recovery. This distinction in design ensured that the study could effectively compare the intervention effects of the two different training philosophies under similar conditions of training duration and intensity.

### 2.3 Experimental test indicators

At different time points of the experiment (0, 4 and 8 weeks), the relevant indicators were determined.

1. FMS functional motion screening: it is composed of seven basic test actions reflecting human flexibility and stability (12), including: over the top squat, hurdle step, straight lunge squat, shoulder flexibility, active straight-leg raise, trunk stability push ups and rotary stability; The test is carried out in the order of 7 actions. After each action, the score is given according to the scoring standard. According to the quality of the action completed by the FMS subject, 4 grade standards are given for evaluation. Pain during the test, inability to complete the whole action, ability to complete the action but low quality of action completion, and ability to complete the whole action according to the standard were recorded as 0, 1, 2, and 3 points, respectively. All tests were completed by one person.
2. Physical fitness test indicators: Based on the new version of the national student physical health standard of China, the selected contents are as follows: 50 m running, 800/1,000 m running, sitting forward bending, standing long jump, vital capacity. The speed, endurance, flexibility, sensitivity, balance, jumping ability and coordination ability of the experimental subjects were evaluated and tested, respectively.

### 2.4 Statistical treatment

To guarantee adequate statistical power for testing the core hypothesis, we did *a priori* power analysis with GPower (Version 3.1) before the experiment. The analysis focused on the time-group interaction effect in repeated measures ANOVA to determine the required sample size. We drew on the conventions in related fields for the parameters. They included a medium effect size (*f* = 0.25), *α* = 0.05, and power (1 − β) = 0.80. Other parameters were two groups, three repeated measures, and an estimated correlation among repeated measures of 0.5. Given these, GPower indicated a minimum of 34 participants. Our study recruited 40 valid participants, satisfying and slightly exceeding the calculated requirement. This shows the study has sufficient statistical power to test the main hypothesis.

## 3 Results

### 3.1 Comparative analysis of FMS scores after 8-week functional training

Functional training for adolescents significantly improves motor control and balance, particularly in integrating complex movement patterns1. The Functional Movement Screen (FMS) serves as a critical tool to assess these changes, enabling the identification of movement deficiencies and providing evidence-based guidance for interventions. This section details the comparative changes in FMS scores for both groups following the 8-week intervention.

#### 3.1.1 Temporal changes in overhead squat, hurdle step, and in-line lunge scores

Following the intervention, both the control and experimental groups exhibited progressive improvements in their Overhead Squat and Hurdle Step scores. For the In-Line Lunge, the experimental group showed sustained improvement, whereas the control group’s performance initially increased but then declined. The repeated measures ANOVA results are presented in Table 3. A significant and large main effect of time was observed for all three movement patterns (overhead squat: *F* = 15.618, *p* < 0.01, *η*^2^ = 0.34; Hurdle step: *F* = 16.732, *p* < 0.01, *η*^2^ = 0.32; In-Line Lunge: *F* = 7.724, *p* = 0.001, *η*^2^ = 0.17), indicating that training over 8 weeks led to significant overall improvements across all participants. However, the time × group interaction effect, which determines the difference between the training modalities, was only significant for the In-Line Lunge (*F* = 3.586, *p* = 0.034), with a medium effect size (*η*^2^ = 0.10). This demonstrates that the functional training program was significantly more effective than traditional training specifically for improving In-Line Lunge performance. No significant differences between the groups’ improvement rates were found for the Overhead Squat (*p* = 0.084) or the Hurdle Step (*p* = 0.264).

**Table 3:** Comparative scores of FMS movement patterns across timepoints (units: points).

Movement	Group	Week 0	Week 4	Week 8	F_(CON-EXP)_, *p*, *η*^2^	F_(EXP0-4-8)_, *p*, *η*^2^
Overhead squat	Control	1.87 ± 0.990	2.20 ± 0.775	2.40 ± 0.737	0.081, *p* = 0.084, 0.002	15.618, *p* < 0.01, 0.34
	Experimental	2.40 ± 0.737	2.67 ± 0.488	2.93 ± 0.258		
Hurdle step	Control	1.87 ± 0.743	2.07 ± 0.799	2.27 ± 0.799	1.36, *p* = 0.264, 0.001	16.732, *p* < 0.01, 0.32
	Experimental	2.00 ± 0.655	2.20 ± 0.561	2.67 ± 0.488		
In-line lunge	Control	1.87 ± 0.64	2.07 ± 0.594	2.00 ± 0.000	3.586, *p* = 0.034, 0.10	7.724, *p* = 0.001, 0.17
	Experimental	2.20 ± 0.561	2.53 ± 0.516	2.87 ± 0.352		

F_(CON-EXP)_ refers to the comparison between the experimental group and the control group, while F_(EXP0-4-8)_ refers to the comparison between the experimental group before and after the experiment.

#### 3.1.2 Shoulder mobility score changes

Both groups showed modest gains in shoulder mobility scores over the 8-week period (Table 4). The ANOVA results confirmed that there was no significant main effect of time (*F* = 2.390, *p* = 0.116) nor a significant time × group interaction effect (*F* = 0.341, *p* = 0.656). These findings suggest that neither training program provided a sufficient stimulus to induce statistically significant improvements in shoulder mobility.

**Table 4:** Shoulder mobility score progression.

Group	Week 0	Week 4	Week 8	F_(CON-EXP)_, *p*, *η*^2^	F_(EXP0-4-8)_, *p*, *η*^2^
Control	2.67 ± 0.488	2.73 ± 0.458	2.73 ± 0.458	2.390, *p* = 0.116, 0.002	0.341, *p* = 0.656, 0.001
Experimental	2.60 ± 0.507	2.67 ± 0.488	2.73 ± 0.458		

F_(CON-EXP)_ refers to the comparison between the experimental group and the control group, while F_(EXP0-4-8)_ refers to the comparison between the experimental group before and after the experiment.

#### 3.1.3 Changes in active straight-leg raise (ASLR), trunk stability push-up, and rotary stability scores

As documented in Table 5, the experimental group demonstrated sustained gains in ASLR, while the control group showed transient improvement followed by a decline. Both groups exhibited progressive improvements in the Trunk Stability Push-up and Rotary Stability tests. The statistical analysis revealed highly significant main effects of time for all three tests, with large effect sizes (η^2^ ranging from 0.39 to 0.45). More importantly, a significant time × group interaction effect was found for all three components: ASLR (*F* = 6.870, *p* = 0.002, *η*^2^ = 0.16), Trunk Stability Push-up (*F* = 3.800, *p* = 0.028, *η*^2^ = 0.11), and Rotary Stability (*F* = 6.213, *p* = 0.004, *η*^2^ = 0.19). This indicates that the functional training intervention was significantly superior to traditional training in enhancing core stability, hip mobility, and coordinated control of the trunk and limbs.

**Table 5:** Temporal changes in additional FMS components.

Test	Group	Week 0	Week 4	Week 8	F_(CON-EXP)_, *p*, *η*^2^	F_(EXP0-4-8)_, *p*, *η*^2^
Active straight-leg raise	Control	1.73 ± 0.458	2.07 ± 0.258	2.00 ± 0.378	22.522, *p* < 0.001, 0.39	6.870, *p* = 0.002, 0.16
	Experimental	2.00 ± 0.535	2.53 ± 0.516	2.93 ± 0.258		
Trunk stability push-up	Control	1.73 ± 0.704	2.07 ± 0.458	2.20 ± 0.414	24.200, *p* < 0.001, 0.42	3.800, *p* = 0.028, 0.11
	Experimental	1.93 ± 0.704	2.33 ± 0.488	2.93 ± 0.258		
Rotary stability	Control	1.73 ± 0.458	2.00 ± 0.378	2.13 ± 0.352	30.402, *p <* 0.001, 0.45	6.213, *p* = 0.004, 0.19
	Experimental	1.80 ± 0.676	2.40 ± 0.507	2.87 ± 0.352		

F_(CON-EXP)_ refers to the comparison between the experimental group and the control group, while F_(EXP0-4-8)_ refers to the comparison between the experimental group before and after the experiment.

### 3.2 Effects of 8-week functional training on physical fitness indicators

#### 3.2.1 Baseline equivalence between groups

An independent samples t-test was conducted on the baseline data for all physical fitness metrics. As shown in Table 6, no significant differences were found between the experimental and control groups at the start of the study (*p* > 0.05 for all indicators), confirming cohort comparability for the intervention.

**Table 6:** Baseline physical fitness parameters.

Parameter	Experimental	Control	t	*p*-value
50-m sprint (s)	7.445 ± 0.427	7.50 ± 0.598	−0.673	0.506
1,000-m run (min:s)	4.122 ± 0.672	4.128 ± 0.538	−0.029	0.978
Sit-and-reach (cm)	13.490 ± 6.011	13.207 ± 4.912	0.149	0.883
Standing long jump (cm)	217.05 ± 14.745	218.13 ± 19.912	0.481	0.634
Vital capacity (ml)	4512.55 ± 753.49	4439.00 ± 782.66	1.045	0.804

#### 3.2.2 Physical fitness improvements post-intervention

Following the 8-week intervention, both groups demonstrated progressive enhancements across all measured fitness metrics (Table 7). The ANOVA results indicated a significant main effect of Experimental intervention for all five parameters (*p* = 0.014), with large effect sizes (η^2^ ranging from 0.13 to 0.38), confirming that both training programs led to overall improvements in physical fitness. Analysis of the time × group interaction effects revealed that the functional training group achieved significantly greater gains in the 50-m sprint (*F* = 7.689, *p* = 0.045, *η*^2^ = 0.10), 1,000-m run (*F* = 0.233, *p* = 0.048, *η*^2^ = 0.08), sit-and-reach (*F* = 5.931, *p* = 0.002, *η*^2^ = 0.10), standing long jump (*F* = 2.224, *p* = 0.032, *η*^2^ = 0.09), and vital capacity (*F* = 19.402, *p* < 0.001, *η*^2^ = 0.26). The particularly large effect size for the improvement in vital capacity suggests a substantial and practically significant advantage for the functional training group in this area.

**Table 7:** Temporal changes in physical fitness metrics.

Parameter	Group	Week 0	Week 4	Week 8	F_(CON-EXP)_, *p*, *η*^2^	F_(EXP0-4-8)_, *p*, *η*^2^
50-m sprint (s)	Control	7.50 ± 0.598	7.46 ± 0.657	7.36 ± 0.642	7.689, *p* = 0.045, 0.1	4.884, *p* < 0.001, 0.17
	Experimental	7.445 ± 0.427	7.275 ± 0.325	7.035 ± 0.241		
1,000-m run (min:s)	Control	4.128 ± 0.538	4.0167 ± 0.569	3.9593 ± 0.539	0.233, *p* = 0.048, 0.08	6.955, *p* < 0.001, 0.38
	Experimental	4.122 ± 0.672	3.933 ± 0.569	3.580 ± 0.543		
Sit-and-reach (cm)	Control	13.207 ± 4.912	13.473 ± 4.929	13.727 ± 4.924	5.931, *p* = 0.002, 0.10	7.200, *p* = 0.014, 0.13
	Experimental	13.490 ± 6.011	14.285 ± 5.754	19.495 ± 4.893		
Standing long jump (cm)	Control	218.13 ± 19.912	220.93 ± 22.330	223.47 ± 21.804	2.224, *p* = 0.032, 0.09	11.302, *p* < 0.001, 0.29
	Experimental	217.05 ± 14.745	223.75 ± 14.48	236.10 ± 11.016		
Vital capacity (ml)	Control	4,439 ± 782.66	4239.2 ± 783.43	4339.27 ± 776.09	19.402, *p* < 0.001, 0.26	11.302, *p* < 0.001, 0.28
	Experimental	4512.5 ± 753.49	5000.05 ± 721.54	5678.85 ± 674.08		

F_(CON-EXP)_ refers to the comparison between the experimental group and the control group, while F_(EXP0-4-8)_ refers to the comparison between the experimental group before and after the experiment.

## 4 Discussion

The findings of this study strongly support that for college—aged males, functional training (FT) is more effective than traditional training in simultaneously enhancing functional movement quality and overall physical fitness. This advantage stems from FT’s focus on neuromuscular integration and cooperation, unlike the isolated strengthening of physical components in traditional training. By replicating multi-joint, multi-plane movements from real life and sports, FT optimizes kinetic chain transmission, core stability, and proprioceptive feedback. The following discussion explores the mechanisms and significance of these findings from neurophysiological, biomechanical, and practical angles.

Delving into the underlying mechanisms (mechanism elucidation), the superiority of FT can be explicated from neurophysiological, metabolic adaptation, and biomechanical perspectives. At the neural level, FT, with its emphasis on motor control under varied environmental conditions and augmented proprioceptive input, may enhance muscle spindle sensitivity to changes in muscle length and tension through increased *γ*-efferent drive (13). This, in turn, optimizes motor unit recruitment efficiency and intermuscular coordination, which is crucial for improving performance in FMS tests requiring high levels of balance and coordination, such as the in-line lunge and rotary stability. Physiologically, the explosive power and intermittent high-intensity elements frequently incorporated into FT (e.g., medicine ball slams followed by sprints, multi-directional speed drills) may not only promote an adaptive increase in the proportion of fast-twitch muscle fibers (Type IIa) (14), thereby enhancing anaerobic power (reflected in 50-m sprint and standing long jump performance), but also effectively elevate the individual’s lactate threshold (15), on functional circuit training and metabolic adaptations. This delays fatigue onset and consequently improves endurance in events such as the 1,000-m run. From a biomechanical standpoint, FT places significant emphasis on establishing core “stiffness” and efficient force transmission. Systematic core stability exercises enhance the pre-activation and co-contraction of deep trunk musculature, thereby improving spinal stability during dynamic tasks. This aligns with the “core stiffness hypothesis” proposed by Fallah (16), which posits that a stable core facilitates more effective axial load transmission and dissipation, providing a solid foundation for rapid and forceful limb movements, proving particularly beneficial for improving trunk stability push-up performance and overall movement efficiency.

Notably, functional training in this study did not significantly improve shoulder mobility, overhead squats, and hurdle steps. This highlights the “specificity principle” in training: even comprehensive functional training may not match the unique demands of specific movements. These tests require high joint mobility and complex motor control, often limited by the “weakest link” in the kinetic chain. Specifically, overhead squats are restricted by thoracic spine extension and ankle dorsiflexion; shoulder mobility depends on thoracic spine and shoulder complex range-of-motion; hurdle steps need hip dynamic dissociation and single-leg stability. While the functional program here enhanced strength and core stability, it lacked targeted exercises for these mobility issues. For example, it focused on shoulder girdle stability over flexibility, and lower—limb strength over ankle and thoracic spine mobility. This result does not negate functional training’s value but suggests a constructive improvement: functional training is effective as a general approach, but for individual comprehensive development, it’s crucial to combine general training with targeted corrective exercises based on diagnostic tools like the FMS. This combination is the key to better training results.

Based on the preceding analysis, the following practical recommendations and future directions are proposed to more effectively enhance the physical health of university students (solution proposal). It is recommended to develop a “Periodized Fusion Training Model” (17). This model would strategically integrate FT with traditional resistance training and sport-specific skill training across different training cycles. For instance, a preparatory phase could focus on FT to improve movement patterns, activate the core, and prevent injuries; a developmental phase could incorporate a greater proportion of traditional resistance training to build foundational strength and muscle mass; and a competitive or pre-testing phase could re-emphasize FT to optimize sport-specific performance and adaptation. Additionally, the incorporation of wearable Inertial Measurement Unit (IMU) sensor technology is advocated for objective FMS movement quantification (18), real-time monitoring of training quality (e.g., core stability, force production timing), and provision of immediate feedback, thereby enhancing training precision and efficacy. Future research should further explore the long-term effects of varying dosages and combinations of FT protocols on specific physiological and biomechanical parameters, and investigate their applicability and specificity across diverse student populations, including different genders and varying baseline fitness levels.

This study, despite its rigorous design and significant findings, possesses several limitations that warrant consideration. Firstly, the sample was comprised of ordinary male university students from a single institution, which may limit the generalizability of our findings to other populations, such as female students, trained athletes, individuals of different age groups, or those from diverse cultural backgrounds. While the sample size (*N* = 40) was sufficient to detect significant differences for the primary outcomes, larger cohorts would provide greater statistical power for more nuanced subgroup analyses and enhance the external validity of the results. Secondly, while the functional training program proved effective, its specific design might not have optimally targeted all components of the FMS; for instance, the less pronounced improvements in shoulder mobility, the overhead squat, and the hurdle step suggest that these areas might require more specialized or supplementary exercises. Furthermore, although we elucidated potential neurophysiological, metabolic, and biomechanical mechanisms, direct measures of these adaptations (e.g., electromyographic analysis of muscle recruitment patterns, muscle biopsies for fiber type shifts, direct assessment of lactate threshold, or advanced neuroimaging of cortical adaptations) were not undertaken in this study; thus, the proposed mechanisms remain inferential. Thirdly, certain external variables were not strictly controlled. While participants were instructed to maintain their usual lifestyle and avoid other structured training, precise monitoring of dietary intake and daily physical activity levels outside the intervention was not implemented, potentially introducing unmeasured variability. Moreover, the “traditional training” protocol served as a control but its specific composition, while standard, may vary across institutions, potentially influencing comparative interpretations. Finally, this study did not incorporate a long-term follow-up assessment, which would be valuable for determining the persistence of the observed training effects and the sustainability of the improvements in FMS scores and physical fitness. The study also did not directly track injury incidence, thus precluding direct conclusions about the FT program’s efficacy in injury prevention, although improved FMS scores are often associated with reduced injury risk.

## 5 Conclusion

In conclusion, this study demonstrates that an 8-week functional training program significantly enhances functional movement patterns, as assessed by the Functional Movement Screen, and improves a comprehensive range of physical fitness indicators—including speed, power, endurance, and flexibility—in ordinary male university students when compared to a traditional training regimen. Specifically, functional training yielded superior improvements in dynamic stability, core strength, and coordinated movement capabilities. These findings underscore the efficacy of functional training as a potent intervention strategy for concurrently improving movement quality and overall physical health in this demographic. The results advocate for the broader incorporation of well-designed functional training principles into university physical education curricula and student wellness initiatives. Future research should focus on optimizing functional training program variables for diverse populations, directly measuring underlying physiological and biomechanical adaptations, and exploring the long-term benefits and injury prevention potential through longitudinal studies, potentially leveraging emerging technologies such as IMU sensors and individualized training models.

## Data Availability

The raw data supporting the conclusions of this article will be made available by the authors, without undue reservation.
